# Magnetic resonance imaging findings within the posterior and lateral columns of the spinal cord extended from the medulla oblongata to the thoracic spine in a woman with subacute combined degeneration without hematologic disorders: a case report and review of the literature

**DOI:** 10.1186/1752-1947-5-166

**Published:** 2011-04-27

**Authors:** Samira Rabhi, Mustapha Maaroufi, Hajar Khibri, Faouzy Belahsen, Siham Tizniti, Rhizlane Berrady, Wafaa Bono

**Affiliations:** 1 Department of Internal Medicine, Hassan II University Hospital, Fez, Morocco; 2 Department of Radiology, Hassan II University Hospital, Fez, Morocco; 3 Department of Neurology, Hassan II University Hospital, Fez, Morocco

## Abstract

**Introduction:**

Subacute combined degeneration of the spinal cord is a rare cause of demyelination of the dorsal and lateral columns of the spinal cord and is a neurological complication of vitamin B_12 _deficiency. Subacute combined degeneration without anemia or macrocytosis is rare.

**Case presentation:**

We present a case of cobalamin deficiency in a 29-year-old Moroccan woman who presented with subacute combined degeneration without evidence of anemia or macrocytosis. Magnetic resonance imaging of the spinal cord demonstrated abnormal hyperintense signal changes on T2-weighted imaging of the posterior and lateral columns from the medulla oblongata to the thoracic spine. A diagnosis of subacute combined degeneration of the spinal cord was considered and confirmed by low serum cobalamin. The patient was treated with vitamin B_12 _supplements and showed improvement in her clinical symptoms.

**Conclusion:**

Physicians should diagnose subacute combined degeneration in patients early by having a high index of suspicion and using diagnostic tools such as magnetic resonance imaging.

## Introduction

Vitamin B_12 _deficiency usually presents with various hematological, gastrointestinal and neuropsychiatric manifestations. Commonly seen neuropsychiatric manifestations include myelopathy, neuropathy, dementia, neuropsychiatric abnormalities and, rarely, optic nerve atrophy. Subacute combined degeneration (SCD) of the spinal cord is an uncommon cause of myelopathy but is the most frequent clinical manifestation of vitamin B_12 _deficiency [1]. As anemia is a common early symptom leading to the diagnosis of vitamin B_12 _deficiency, neurological symptoms have often been considered to be late manifestations and typically occur after the development of anemia [2]. We present the magnetic resonance imaging (MRI) scans of a patient with SCD involving the lateral and posterior columns extended to segments of spinal cord and without anemia or macrocytosis.

## Case presentation

A 29-year-old Moroccan woman came to our institution complaining of numbness and tingling of four months' duration in both lower limbs, with unsteady gait and easy falling and urine incontinence. The patient's background and history did not reveal preexisting diabetes mellitus, alcohol addiction, vegetarian food preference or gastrointestinal symptoms. She did not mention any fever, night sweats or itching. On physical examination, her temperature was 37.4°C, her pulse was 80 beats/minute and her blood pressure was 120/83 mmHg. She had no pallor or icterus and no lymphadenopathy, edema, splenomegaly or hepatomegaly. On neurological examination, her deep tendon reflexes were hyperactive in the upper and lower extremities. Babinski's sign, Romberg's sign and Lhermitte's sign were present. Vibration and joint position sense examination were evaluated as decreased. However, there was no decrease in light touch sensation. Her laboratory examination values were unremarkable: white blood cell count 8,500/mm^3^, hemoglobin 13 g/dL, mean corpuscular volume 97/μ^3^, platelets 225,000/mm^3 ^and thyroid-stimulating hormone 1.5 mU/L.

**Figure 1:**
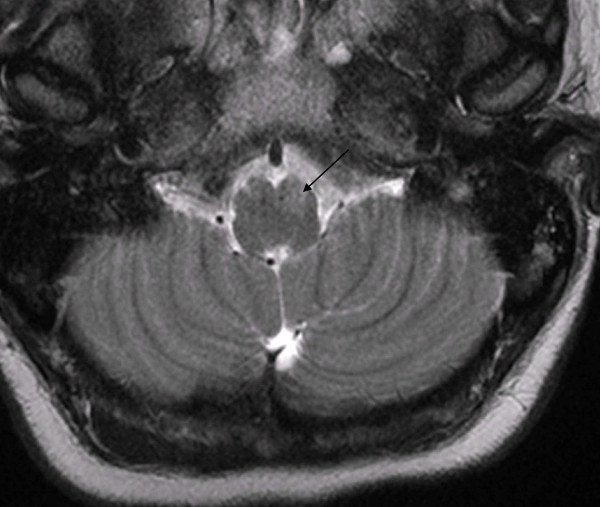
**Transverse T2-weighted magnetic resonance imaging (MRI) scan of the posterior cerebral fossa showing symmetric signal intensity within the medulla oblongata before treatment**.

**Figure 2:**
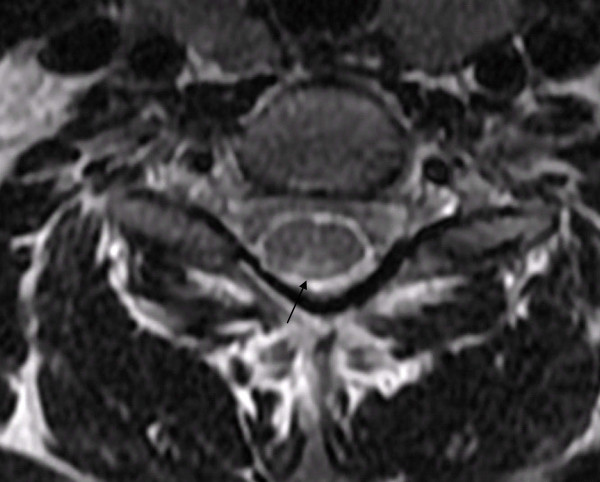
**Transverse T2-weighted MRI scan of the cervical spinal cord at the C2 level demonstrating bilateral symmetric signal intensity within the dorsal columns (inverted V sign) before treatment**.

**Figure 3:**
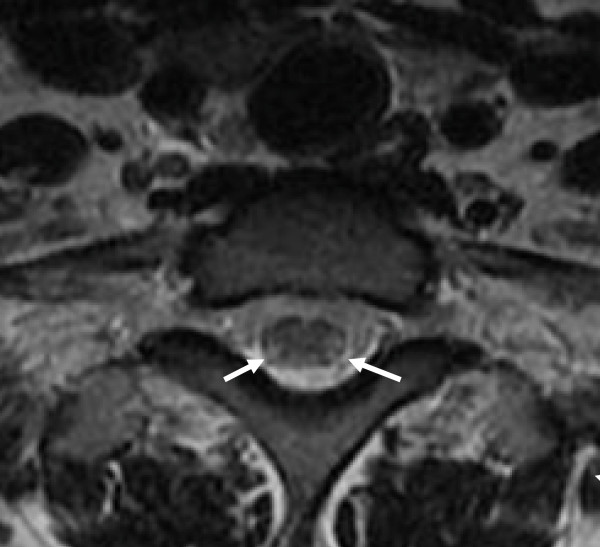
**Transverse T2-weighted MRI scan of the cervical spinal cord at the C7 level demonstrating symmetric signal intensity within the lateral and dorsal columns before treatment**.

**Figure 4:**
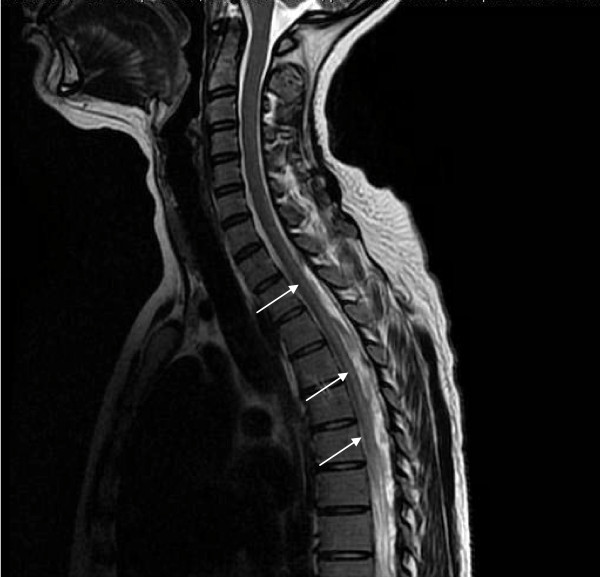
**Sagittal T2-weighted MRI scan showing the dorsal spinal cord with hyperintensity involving the posterior and lateral columns before treatment**.

**Figure 5:**
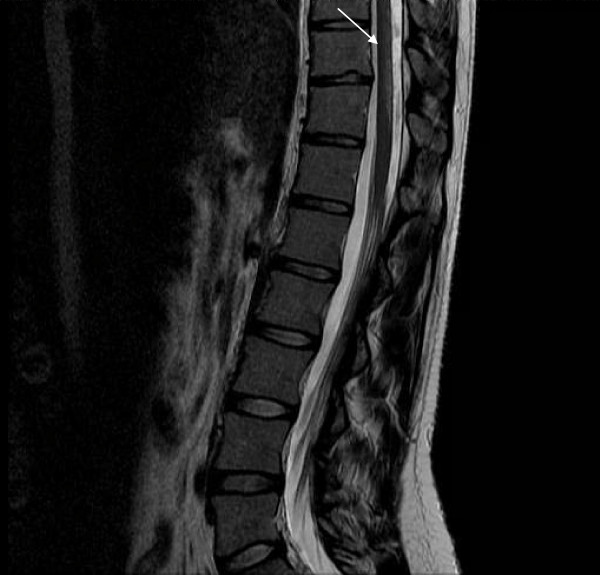
**Sagittal T2-weighted MRI scan showing an area of hyperintensity involving the bilateral posterior and lateral columns of the thoracic and lumbar junction before treatment**.

The initial MRI examination of the cervical and dorsal spine was performed using a 1.5-T unit and showed an area of hyperintensity involving the dorsal and lateral columns from the medulla oblongata (Figure 1) to the thoracic spine (Figure 2, Figure 3 and Figure 4) on T2-weighted images. This area was not enhanced after the addition of gadolinium. The axial images revealed involvement of the posterior and lateral columns bilaterally (Figures 4 and 5), which was highly suggestive of SCD. The serum vitamin B_12 _level was collapsed to 25 pg/mL (normal range, 180 to 914 pg/mL), and her serum vitamin E level was normal.

**Figure 6:**
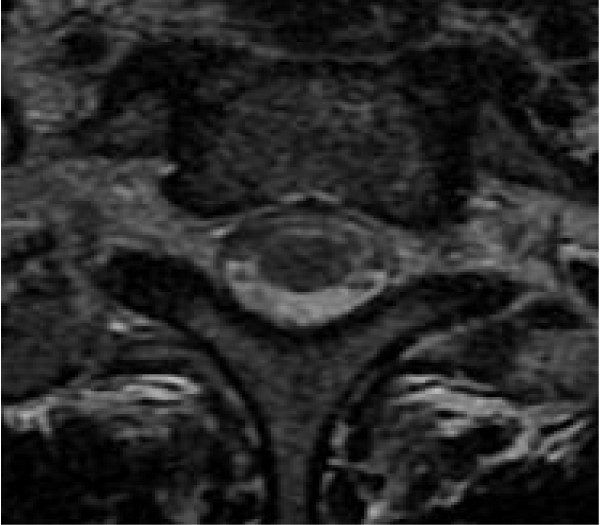
**Transverse T2-weighted MRI scan of the cervical spinal cord at the C7 level with normal signal intensity of the lateral and dorsal columns after treatment**.

Bone marrow aspirates showed a medullary megaloblastosis. A Schilling test was not available. Upper gastrointestinal examination revealed fundic atrophic gastritis. Parietal cell antibodies and anti-intrinsic factor were positive. The neurological symptoms totally disappeared two months after intramuscular supplementation of vitamin B_12 _1,000 μg daily for one week, then weekly for two weeks, and then monthly. The MRI scan abnormalities were significantly improved (Figure 6).

## Discussion

We describe new MRI findings of the spinal cord in SCD revealing vitamin B_12 _deficiency without anemia and or macrocytosis. The high-signal intensity, T2-weighted MRI findings within the posterior and lateral columns extended from the medulla oblongata to the thoracic spine.

The myelopathy of vitamin B_12 _deficiency (or SCD) is characterized neuropathologically by degeneration of myelin and axonal loss [3]. It is clear now that the neuropathological lesions in SCD are due to overproduction of myelinolytic tumor necrosis factor α (TNF-α) and to the reduced synthesis of the two neurotrophic agents epidermal growth factor (EGF) and interleukin-6. This deregulation of the balance between TNF-α and EGF synthesis is induced by cobalamin deficiency [4].

Neuropathological studies show the main lesions to be in the posterior and lateral columns, predominantly in the upper thoracic and midthoracic regions [5]. The MRI findings of the spinal cord in SCD are high-signal intensity on T2-weighted images within the posterior or lateral columns. Brain lesions of vitamin B_12 _deficiency over the medulla oblongata, pons, mesencephalon and crus cerebelli have also been reported [6]. SCD can also result from common variable immunodeficiency syndrome, paraneoplastic malabsorption, folate deficiency, acute monoblastic leukemia and nitrous oxide anesthesia [7].

The main symptoms of SCD are paresthesia, stiffness, numbness or tingling of the limbs; sensory ataxia; and impaired vibration and joint position sensation. Spastic paraparesis may develop if SCD is left untreated. Babinski's sign may be present, and the deep tendon reflexes are variable [8]. If these symptoms are associated with macrocytic anemia, the possibility of SCD should be strongly considered. Usually, vitamin B_12 _deficiency is detected on the basis of hematological abnormalities such as macrocytic megaloblastic anemia or macrocytosis, but it was noted to be associated with only neuropsychiatric abnormalities in 28% of one population studied [2]. The hematologic abnormalities of vitamin B_12 _deficiency (macrocytic anemia) may develop after neurologic abnormalities. Some patients with SCD might have minimal symptoms without hematologic abnormalities initially, such as acroparesthesia and Lhermitte's sign only. At this moment, in the early stage, in addition to blood vitamin B_12 _and homocysteine levels, spinal MRI may be a good diagnostic tool [9]. Once the diagnosis of SCD is suspected, treatment with vitamin B_12 _injection should be started as early as possible to avoid irreversible neurologic damage. Improvement in myelopathy may occur if vitamin B_12 _therapy is started early in the course of the disease. The resolution of the MRI changes in our case correlated well with the clinical improvement [8,10].

## Conclusion

We report a case of an adult with SCD with new MRI findings from the medulla oblongata to the thoracic spine with high signal intensity on T2-weighted images within the posterior or lateral columns and without hematologic disorders. There have been few cases reported in the literature with extended lesions over several segments of the spinal cord. Complete recovery from the disease was not observed in the previous reports. Patients with SCD should be diagnosed early by their treating physicians having a high index of suspicion and using diagnostic tools such as MRI.

## Consent

Written, informed consent was obtained from the patient for publication of this case report and accompanying images. A copy of the written consent is available for review by the Editor-in-Chief of this journal.

## Competing interests

The authors declare that they have no competing interests.

## Authors' contributions

SR wrote the manuscript with comments and revision. MM prepared the figures. All authors read and approved the final manuscript.
